# Africa is on the verge of a major health crisis and the need for nutrition and health surveys is imperative

**DOI:** 10.11604/pamj.2018.30.173.15340

**Published:** 2018-06-26

**Authors:** Seth Selorm Klobodu, Sarah Kessner, Levi Johnson

**Affiliations:** 1 Department of Nutrition and Food Science, California State University, Chico, USA

**Keywords:** Non-communicable diseases, obesity, overweight, nutrition and health surveys

## Abstract

The African region may be reaching a tipping point for a major health crisis. Over the last few decades for example, there has been a significant increase in the prevalence of obesity and associated comorbidities. This alarming trend is attributed to the rapid urbanization, increase in income, and unhealthy lifestyles among many others. Most importantly, managing and treating non-communicable diseases (NCDs) is expensive and proving to be beyond the reach of ordinary Africans. Interestingly, Africa has not fully eradicated undernutrition particularly micronutrient malnutrition especially among women and children. Additionally, HIV/AIDs, malaria, diarrheal diseases and other preventable infectious diseases continue to pose serious threat to the health of the populace. The management of NCDs is challenging and costly as most healthcare systems on the continent are largely built for the provision of curative care for communicable diseases. This article proposes the institution of nutrition and health surveys in individual countries to monitor trends. We believe this approach, in addition to addressing lifestyle and behavioral factors, may be effective in curbing the rising phenomenon of obesity and NCDs.

## To the editors of the Pan African Medical Journal

In the last few decades, Africa has seen a significant rise in the prevalence of obesity and associated non-communicable diseases (NCDs). At the same time, the region has witnessed the proliferation of major fast food companies. It is therefore not surprising that recent publications in the New York Times (NYT) and Quartz have highlighted the association between the explosion of major fast food companies in Africa and the rapid rise in obesity as well as NCDs [1-3]. The NYT and Quartz rightly identified the excessive intake of high-energy foods particularly fast foods, processed foods high in salt, sugar and fat, decrease in physical activity, and an emerging shift in genetic predisposition as probable culprits. However, the entire picture is complex and addressing this pervasive problem requires an innovative and holistic approach. The World Health Organization's (WHO) recent report is less than encouraging to say the least. The report noted that key risk factors for NCDs are widespread, jeopardizing the future health of Africans [4]. For example, the prevalence of overweight ranged from a low of 12% in Madagascar to a high of 60% in Ghana and Seychelles [4]. Similarly, the prevalence of obesity has been reported to range from 2% in Madagascar to 27% in Ghana [4]. A high percentage of adults living in this region are hypertensive [5]. The African countries with some of the highest rates of hypertension are Seychelles (40%), Cabo Verde (39%), Sao Tome and Principe (39%), Ghana (37%) and Niger (36%) [4]. Diabetes and elevated blood lipid levels are also emerging public health concerns across the continent. Physical inactivity is widespread especially as educated, middle class Africans are increasingly engaged in sedentary and desk-bound jobs [4]. This unprecedented phenomenon, if not curbed, will have dire consequences on the economy and the already ill-equipped health systems in Africa. As members of the public health nutrition community, we are equally concerned with the shift in dietary consumption from traditional, nutrient-dense African foods to a more “Westernized diet“- one that is very energy-dense, high in both sugar, salt and saturated fat. This comes on the back of the expanding economies resulting in rapid urbanization and the rise in middle-income families. This change in status comes with access to more “disposable income“ which may be spent on “luxury“ foods such as fast food, which is considered a symbol of affluence in certain African cultures. It is interesting to note that some cultures have traditionally viewed being overweight as a sign of “good living“. This cultural perspective may be because of the fact that only the wealthy could afford food and be well-fed in times past when literacy levels were relatively low, poverty was rampant, when food was not nearly as abundant or accessible and famine was widespread on the continent. Only a few decades past, Western foods such as fried chicken, French fries, and processed desserts were not a daily delicacy, but were rather reserved for special occasions like religious holidays and New Year's celebrations. It was also rare to hear young adults die of heart disease or even have hypertension in comparison to now. Current WHO statistics indicate that schoolchildren in this region are not engaging in the recommended amount of physical activity [4]. Besides, they are increasingly being exposed to energy-dense, high-sugar and processed foods as well. It is therefore unsurprising that the prevalence of overweight or obesity among children in Africa has increased from 4 to 9 million between 1990 and 2016 [6]. To address these complex health challenges, we suggest the development of a national program similar to National Health and Nutrition Examination Survey to assess the health and nutritional status of adults and children in each African country. Such a survey is long overdue and could play a vital role in the monitoring the health and nutrition of Africans. Currently, very few countries have implemented such surveys. South Africa readily comes to mind; however, their Health and Nutrition Examination Survey is recent.

## Conclusion

We believe such surveys will generate critical information necessary for policy development, planning, and evaluation. In order to achieve this, there needs to be political will and also calls for multidisciplinary collaboration involving diverse disciplines including culture, anthropology, psychology, and epidemiology, nutrition, clinical, and economic research to properly inform the design of this national program. This may also include possible interventions that address the needs of people with or at risk of NCDs. We believe these steps are critical to help the economic development of Africa.

## Competing interests

The authors declare no competing interest.
